# Selenium to selenoproteins – role in COVID-19

**DOI:** 10.17179/excli2021-3530

**Published:** 2021-04-16

**Authors:** Sojit Tomo, Gangam Saikiran, Mithu Banerjee, Sushmita Paul

**Affiliations:** 1 Department of Biochemistry, All India Institute of Medical Sciences, Jodhpur, India; 2 Indian Institute of Technology, Jodhpur, India

**Keywords:** COVID-19, selenium, selenoproteins, virally encoded selenoproteins

## Abstract

The disruption of antioxidant defense has been demonstrated in severe acute respiratory syndrome due to SARS-CoV infection. Selenium plays a major role in decreasing the ROS produced in response to various viral infections. Selenoprotein enzymes are essential in combating oxidative stress caused due to excessive generation of ROS. Selenium also has a role in inhibiting the activation of NF-κB, thus alleviating inflammation. In viral infections, selenoproteins have also been found to inhibit type I interferon responses, modulate T cell proliferation and oxidative burst in macrophages, and inhibit viral transcriptional activators. Potential virally encoded selenoproteins have been identified by computational analysis in different viral genomes like HIV-1, Japanese encephalitis virus (JEV), and hepatitis C virus. This review discusses the role and the possible mechanisms of selenium, selenoproteins, and virally encoded selenoproteins in the pathogenicity of viral infections. Identification of potential selenoproteins in the COVID 19 genome by computational tools will give insights further into their role in the pathogenesis of viral infections.

## Introduction

Coronavirus disease (COVID-19) is a respiratory disease caused by Severe Acute Respiratory Syndrome coronavirus 2 (SARS-CoV-2) which is a successor of SARS CoV. This new type of coronavirus was first identified in Wuhan (Hubei, China) in December 2019 and later spread across the other countries. In March 2020, WHO declared COVID-19 as a pandemic alerting all countries to take precautions for preventing the spread of the virus (WHO Director-General, 2020[56]). The clinical signs and symptoms of this disease vary from mild presentations (cough, sore throat and fever) to severe manifestations such as pneumonia and Acute Respiratory Distress Syndrome (ARDS) causing death. In viral infections, the uncontrolled production of inflammatory mediators plays a major role in acute lung injury leading to ARDS and subsequent death. These pro-inflammatory cytokines, when released, activate the innate immune cells causing local inflammation and acute lung injury (Gadek and Pacht, 1996[8]). Further, in viral infections, oxidative stress is also thought to be a major contributor to ARDS by increasing the reactive oxygen species (ROS). This favors the activation of inflammatory pathways leading to an overt increase of inflammatory cytokines leading to cytokine storm, hypercoagulability, multiorgan failure, and death.

Selenium is a trace element involved in regulating immune response, oxidative stress, and chronic inflammation. Selenium deficiency has been found to weaken the immune system leading to increased susceptibility to viral infections. Its deficiency also has an effect on anti-oxidant defense leading to further activation of the inflammatory pathways causing tissue damage. Apart from the above-mentioned mechanisms, selenium also interferes with the attachment of the virus to the host by inhibition of disulfide exchange reactions, thus having a protective effect against viral infections (Lipinski, 2015[26]). Disulfide exchange reactions, catalyzed by protein disulfide isomerase enzyme, reduces protein disulfides in the virus glycoprotein. As a result, hydrophobic epitopes are unravelled which can punch hole in the host bilayer membrane and facilitate viral entry into host cell. Selenium in sodium selenite form is capable of inhibiting this protein disulfide isomerase enzyme and prevents viral entry (Lipinski, 2015[26]).

Further, deficiency of selenium has been reported to favor the rate at which virus mutates leading to more virulent strains. In this brief review, we discuss the role of micronutrient selenium in the pathogenesis of viral infections and COVID-19 and the resulting possible role of selenium as adjuvant therapy.

## Oxidative Stress in Viral Infections

Viruses like the influenza virus, adenovirus, respiratory syncytial virus, which have an affinity towards the respiratory tract show various signs and symptoms such as nasal congestion, sore throat, fever progressing to severe acute respiratory distress syndrome. It has been demonstrated that viruses do affect the oxidant status deranging the balance between the prooxidants and antioxidants in the body. These viruses induce the production of ROS-inducing enzymes like nicotinamide adenine dinucleotide phosphate oxidases (NADPH oxidases, Nox) and xanthine oxidase (XO) which disturbs the antioxidant balance (Khomich et al., 2018[20]). This increase in ROS and subsequent oxidative stress by viral infection provides a favorable environment for the replication of the virus thereby lending it survival advantage (Reshi et al., 2014[41]). Antioxidant enzymes such as superoxide dismutase, catalase, peroxiredoxins, and glutathione peroxidases are known to play a major role in ameliorating the oxidative stress caused due to viral infections (Ganesh Yerra et al., 2013[9]). These antioxidant enzymes are regulated by the transcription factor nuclear factor E2-related factor 2 (Nrf2). When the ROS levels are normal, the nrf2 transcription factor is regulated in the cytoplasm by kelch-like ECH-associated protein 1 (keap1) which directs Nrf2 to degradation by the ubiquitin pathway. Increased presence of ROS leads to dissociation of Nrf2 transcription factor from Keap1 protein, allowing movement of Nrf2 into the nucleus leading to increased transcription of antioxidant enzymes. Interestingly, the Nrf2 gene also contains antioxidant response element (ARE) like sequences in its promoter which further increases the expression of antioxidant enzymes by feedback loop mechanism (Kwak et al., 2002[23]). Different viruses tend to have differential activation of the above-mentioned pathways. Augmentation of this pathway is observed in mouse models infected with the influenza virus, whereas RSV leads to the downregulation of this pathway due to proteasomal degradation of nrf2 transcription factor (Kosmider et al., 2012[22]). This downregulation of the nrf2 pathway leads to an increase in oxidative stress and activation of the NF-κB pathway. Pan et al had demonstrated that downregulation of the Nrf2 pathway leads to an increase in cytokine production due to augmentation of the NF-κB pathway (Pan et al., 2012[36]). Simultaneous activation and inhibition of this pathway have been demonstrated in viruses like the HIV virus were Nrf2 pathway is inhibited by the HIV virus and activated by HIV proteins. Despite having concurrent paradoxical effects, the Nrf2 pathway is generally inhibited in HIV infection (Ramezani et al., 2018[40]). Summating, viruses, by modulating the Nrf2 pathway, control oxidative stress and maintain favorable cellular environment for its survival and proliferation.

### Oxidative stress in corona viruses

While there are numerous pieces of evidence of increased ROS and oxidative stress in a multitude of viral diseases, the evidence for the same in SARS-CoV is limited. The increased oxidative stress leading to severe lung injury in severe acute respiratory syndrome is associated with innate immunity and activation of inflammatory signaling pathways such as the NF kappa beta pathway (Van den Brand et al., 2014[53]). *In vitro* experiments have demonstrated an increase in ROS in the cell line infected with SARS-CoV which have been attributed to viral protease (SARS-CoV 3CL_pro_) mediated activation of the NF-κB dependent reporter gene. Viral proteases and its activation of the NF-κB pathway leading to ROS generation has been implicated to have an important role in the pathogenesis of SARS-CoV (Lin et al., 2006[25]). Upregulation of genes relating to oxidative stress in peripheral blood mononuclear cells (PBMC) of convalescent SARS-CoV patients confirms the crucial role of oxidative stress in SARS-CoV patients.

Another SARS-CoV protease protein, 3a, is responsible for the activation of mitochondrial cell death pathways involving activation of the MAPK pathway (Padhan et al., 2008[35]). SARS-CoV can also trigger the TLR4-TRIF-TRAF6 pathway enhancing the severity of lung injury. Oxidized phospholipids produced by macrophages in lung induces overproduction of cytokines via the TLR4-TRIF pathway causing lung injury (Imai et al., 2008[16]). The aforementioned studies point to the pivotal role oxidative stress and inflammation have in the pathogenesis of SARS-CoV infection.


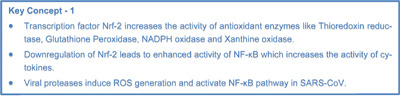


### Inflammation in viral infections 

The increase in ROS caused by oxidative stress in viral infections is followed by activation of inflammatory pathways that lead to an increase in cytokine production and subsequent tissue destruction. The increased ROS in viral infections activate the NF-κB pathway leading to the accumulation of inflammatory cytokines. The excessive production of these inflammatory cytokines culminate in a cytokine storm that causes major damage to the lung tissue (Ye et al., 2020[57]). Animal studies in H1N1 influenza virus infection reported increased nuclear Trx1 (Thioredoxin) which enhanced redox-sensitive transcription of NF-κB contributing to an increase in the inflammatory response and contributes to the severity of the disease (Go et al., 2011[10]). Other respiratory viral infections such as rhinovirus and RSV also have demonstrated augmentation of the NF-κB pathway by increased oxidative stress (Fink et al., 2008[7]). Different viruses act differently on this pathway to gain a survival advantage.

The NF-κB pathway has been known to play an important role in coronavirus infections. Activation of the NF-κB pathway has been demonstrated in the lungs of mice infected with recombinant SARS-CoV virus. Further, when the NF-κB pathway was inhibited, there was a subsequent decrease in the production of inflammatory cytokines although viral titer was not affected (DeDiego et al., 2014[6]). Enhancement of the NF-κB activity was also observed in PBMCs when it was subjected to treatment with recombinant SARS-CoV-S protein. Concurrently, NF-κB inhibitor treatment leads to a decrease in the secretion of inflammatory cytokines confirming the role of this pathway in regulating cytokine levels. The increased levels of proinflammatory cytokines such as IL-8 and IL-6 are suggested to be a product of the interaction of S protein with macrophages and monocytes which leads to activation of NK cells and neutrophils at the site of infection. NF kappa beta pathway plays a major role in inflammation in viral infection by accentuating the production of cytokines such as IL-6, IL-12, TNF alpha that serves to have bearing on adaptive immunity by activating lymphocytes (Kanzawa et al., 2006[19]). Activation of these pathways and overproduction of inflammatory cytokines cause gross inflammation and damage to the lung tissue leading to severe acute respiratory distress (Kanzawa et al., 2006[19]). Treatment involving inhibition of the NF-κB pathway to avoid severe lung injury by using ozone therapy has been postulated by Martínez-Sánchez et al. The modulation of these pathways may have an impact on the cytoprotection and blockage of viral replication (Martínez-Sánchez et al., 2020[31]). Considering the role played by NF-κB in inflammation, inhibitors of the NF-κB pathway are an interesting and exciting area for discovering therapeutic targets for COVID-19.


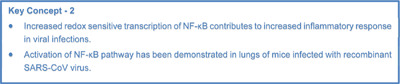


## Selenium and Viral Infections

### Selenium in oxidative stress

Selenium, a nutritionally essential micronutrient, is known for its antioxidant role in mitigating the effects caused by oxidative stress. Being an important component of various antioxidant selenoprotein enzymes like glutathione peroxidase (GPx) and thioredoxin reductase (TrxR), selenium plays an important role in combating oxidative stress caused due to excessive generation of ROS (Tapiero et al., 2003[49]). The antioxidant enzymes like TrxR, apart from maintaining the redox status, also play a crucial role in regulating activities such as cell proliferation, cell death, and immune response activation (Papp et al., 2007[37]). Out of seven GPx enzymes discovered till now, GPx1-4 and GPx6 contain selenium incorporated as selenocysteine in their active sites (Papp et al., 2007[37]). These enzymes act by catalyzing the reduction of hydrogen peroxide and organic hydroperoxides to water or the corresponding alcohol, respectively (Pitts et al., 2014[38]). Other selenoproteins such as selenoprotein K and selenoprotein R have also been found to act as antioxidants in various organs. Further, selenoprotein R catalyzes the reduction of oxidized methionine which is required for the repair of oxidative damage in proteins (Kim and Gladyshev, 2004[21]). Selenoprotein P was found to be protective against oxidative damage in human astrocytes (Steinbrenner et al., 2006[48]). Further, selenoprotein P was found to enhance the activity and expression of glutathione peroxidase in the endothelial cell, thereby protecting the endothelial cells from oxidative damage. The antioxidant properties of numerous selenoproteins have propelled researchers to explore the effect of selenium administration in alleviating oxidative stress. Selenium, in animals, was found to relieve the oxidative stress and immune damage caused due to various factors (Liu et al., 2018[28]). Fenitrothion is a broad-spectrum organophosphorous insecticide that is known to cause oxidative stress by affecting the antioxidant enzyme activities. Hepatoprotective role of selenium was reported in rat liver by decreasing the oxidative stress induced by Fenitrothion (Milošević et al., 2018[32]). Although limited evidence is available regarding selenium administration in humans, intravenous sodium selenite was found to increase serum concentrations of GPx3 and restore antioxidant capacity of lungs in patients with ARDS in a randomized control trial (Mahmoodpoor et al., 2019[30]). The trial, which recruited 40 ARDS patients (20 in each group), demonstrated linear correlation between selenium concentrations and serum concentrations of GPx3 (Mahmoodpoor et al., 2019[30]).

### Selenium and inflammation 

Selenium has been found to play a major role in inflammatory signaling pathways. The inhibition of inflammatory signaling pathways by selenium does have beneficial effects on many diseases. Maehira et al. reported that selenium at physiological levels inhibits activation of the NF-κB transcription factor that encodes genes regulating inflammatory cytokines. Further, the reduction of serum selenium levels has been seen to induce the synthesis of CRP by hepatocytes during the acute phase response (Maehira et al., 2003[29]). Up-regulation of Nrf2 signaling by selenium has been shown to attenuate lung injury induced by sepsis (Kwon et al., 2016[24]). Activation of the Nrf2 pathway leads to the enhancement of glutathione synthesis and downregulation of the Nf-kβ pathway culminating in decreased lung injury. Conversely, the Nrf2 knockdown experiment had shown inhibition of glutathione synthesis and downregulation of the NF-κB pathway. NF-κB transcription factor is the final common step of the regulation for the expression of inflammatory cytokines. The modulation of its activity has been the target for many anti-inflammatory drugs. The effect of selenium on Nrf2 and NF-κB signaling culminates in the regulation of inflammatory cytokines. Selenium supplementations have been found to decrease the production of inflammatory cytokines like IL-6 in cell line studies, animal models, and human studies. Contradicting reports of negative correlation between selenium levels and the inflammatory cytokines like IL-1 and IL-6 have been demonstrated by Mahmoodpoor et al., although they were unable to replicate on a larger sample size (Mahmoodpoor et al., 2019[30]). Summarizing, critically low selenium levels might be responsible for the increased inflammatory response, and selenium supplementations may play an immune-modulatory role by regulating the pathways for the production of inflammatory cytokines.


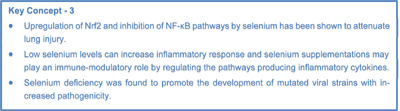


### Selenium in viral infections 

Selenium is postulated to have a pivotal role in combating viral infections. Beck and colleagues, in their pioneering work, have demonstrated a decrease in glutathione peroxidase activity in rats with selenium deficiency which was found to promote the development of viral strains with increased pathogenicity (Beck et al., 2004[3]). The incidence of increased mortality in HIV infected individuals with concurrent selenium deficiency, when compared with infected individuals with adequate selenium, highlights the role of selenium in the progression of disease in viral infections. Experimental studies have also demonstrated that *in vitro* supplementation of selenium stimulates glutathione peroxidase activity and subsequently decreases the activation of HIV Type 1 (Sappey et al., 1994[44]). Selenium has also been demonstrated to play a protective role in individuals infected with the Hepatitis B virus and Hepatitis C virus with disease progression to liver cancer. The increased severity of viral infections in selenium-deficient hosts is postulated to be due to the increased oxidative stress caused due to deficiency of glutathione peroxidase (Beck et al., 1998[4]). This increased oxidative stress culminated in an excessive inflammatory environment due to heightened activation of NF-κB (Beck et al., 2001[5]). Selenium and its deficiency thus have a major role in the development of virulent strains and increased severity in viral infections.

## Selenoproteins and Viral Infections

Various selenoproteins have demonstrated protective as well as pathological functions in viral infections.

Selenoprotein P1 (SEPP1) is observed to be a hepatokine. SEPP1 was found to be involved in the pathogenesis of HCV and HCV induced HCC (Rizk et al., 2019[42]). The levels of hepatic Selenoprotein P (SeP) mRNA (SEPP1 mRNA) and serum SeP are increased in HCV infection (Murai et al., 2019[34]). Retinoic-acid-inducible gene I (RIG-I) is a sensor of viral RNA which has a major role in initiating host immunity against HCV viral infections (Imran et al., 2012[17]; Saito et al., 2008[43]). Activation of type I interferon response has been found to suppress the replication of the HCV virus (Gong et al., 2018[11]). SEPP1 mRNA, by limiting the function of the retinoic-acid-inducible gene I (RIG-I), inhibits type I interferon responses. Since the inhibition of RIG-1 occurs, detection of HCV is difficult hence no immune response is mounted. Direct binding of SEPP1 mRNA to RIG-I inhibits its activity. Further, SEPP1 mRNA knockdown in hepatocytes caused the induction of interferon-stimulated genes and decreased HCV replication (Murai et al., 2019[34]). By SEPP 1 knockdown, there was an unhindered activity of RIG-1 hence good immune response was mounted which kept the HCV virus in control. 

### Association of selenoproteins in immunoprotection, cancer, and homeostasis

Selenoprotein K, in animal studies, has been shown to have a protective effect against West Nile virus infection. West Nile virus-infected selenoprotein K knock-down mice had lower survival compared with that of wild-type controls. Further, Ca^+2^ dependent functions including T cell proliferation and migration, and oxidative burst in macrophages were decreased in cells from selenoprotein K knock-down mice compared with controls (Verma et al., 2011[54]). Selenoprotein M (SELM) is located in the endoplasmic reticulum and contains the redox motif of cysteine-X-X-selenocysteine type. SELM is over-expressed in HCV induced human hepatocellular carcinoma (HCC) cell lines (Guerriero et al., 2014[13]; Guariniello et al., 2014[12]). Selenoprotein P primarily acts as a selenium transporter and was observed to have a role in glucose homeostasis. Selenoprotein P was found to have a crucial role in the development of insulin resistance caused by HCV (Ali et al., 2016[1]).

### Thioredoxin reductase-1 negatively regulates HIV

Thioredoxin reductase-1 (TR1) is a selenium-containing pyridine nucleotide-disulfide oxidoreductase that reduces protein disulfides to free thiols. It has been observed that TR1 negatively regulates the activity of the HIV-1 encoded transcriptional activator, Tat, in human macrophages. TR1 targets two disulfide bonds within the Cys-rich motif which are required for efficient HIV-1 transactivation (Kalantari et al., 2008[18]).


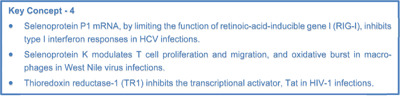


### Virally encoded selenoproteins

Virally encoded selenoproteins are identified because of the presence of a potential UGA stop codon, which could be decoded as selenocysteine. It also requires the presence of a secondary structural selenocysteine insertion sequence (SECIS) which occurs immediately after UGA. The first documented example of virally encoded selenoproteins is MC066L in the molluscum contagiosum virus (MCV) which was revealed in an open reading frame (ORF). The MC066L had homology with human glutathione peroxidase. The MCO66L gene was found to be able to protect cells against UV and peroxide-induced cell death with the enzymatic properties of glutathione peroxidases. This provides a mechanism for the virus to tide over the unfavorable environmental conditions that may prevail in the cell when it infects a cell (Shisler et al., 1998[47]).

Several viruses have been predicted to have selenoproteins embedded in their genome sequence. Identification of potential protein-coding regions containing large numbers of in-frame UGA codons with downstream selenocysteine insertion elements (SECIS) is indicative of potential selenoprotein coding regions. Computational genomic analysis can reveal such potential selenoprotein coding areas in the viral genome. Ebola Zaire, the hemorrhagic fever virus, has coding regions with 17 UGA codons, and several potential SECIS elements. Further, the Ebola Reston strain which does not contain these selenoprotein coding regions are not pathogenic in humans indicating a possible role of these selenoproteins in pathogenesis (Ramanathan and Taylor, 1997[39]).

Similarly, the HIV-1 genome has also been identified to have potential selenoproteins which can be expressed by ribosomal frameshifting and termination suppression (Taylor et al., 1994[52]). Further, Coxsackie B viruses also have been shown to have the existence of highly distinctive glutathione peroxidase (GSH-Px)-related sequences which can lead to potential coding of selenoproteins (Taylor et al., 1997[50]). Comparative sequence analysis has demonstrated the presence of Se-dependent GPx modules in different RNA viruses like HIV-1 and hepatitis C virus, Coxsackievirus B3, HIV-2, and measles virus (Zhang et al., 1999[59]). Apart from GPx modules, a structurally related but functionally distinct selenoprotein gene, related to the iron-binding protein ferredoxin, has been identified on sequence analysis of the Japanese encephalitis virus (JEV) which might play a role in JEV infection and replication (Zhong et al., 2004[60]).


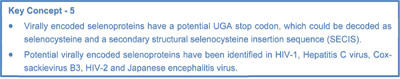


### Mechanisms adopted by viral seleno-proteins which help the virus replicate

Different mechanisms have been postulated regarding the role of the virally encoded selenoproteins in the pathogenesis of viral infections. As a potential viral selenoprotein can consume multiple selenium atoms per molecule, the encoding of protein would lead to scarcity of selenium in the infected host cell leading to ineffective host cell defense against lipid peroxidation and cell membrane destruction. This may contribute to the pathogenesis of viral disease (Ramanathan and Taylor, 1997[39]). A virally encoded selenoprotein like GPx may also help the virus to defend against free-radical-mediated attacks by the immune system (Zhang et al., 1999[59]). A viral GPx might also have a negative effect on viral replication as observed in HIV- 1. But, this might again facilitate virus to tide over the acute phase of cellular reactions by allowing the virus to maintain a low profile during immune activation by defending free-radical-mediated apoptotic attacks (Zhang et al., 1999[59]). 

## Selenium and Coronaviruses

The antioxidant and immunomodulatory role of selenium in coronaviral infections remains unexplored. The association study between the regional selenium status and the outcome of the COVID-19 disease in China reported a higher cure rate and low death rate in the selenium-rich regions when compared with the selenium-deficient regions (Zhang et al., 2020[58]). On assessment of nutritional status of COVID-19 patients, low selenium levels were observed in 42 % of recruited patients (Im et al., 2020[15]). The higher selenium status found in surviving COVID-19 patients when compared with non-survivors points towards the relevant role of selenium in recovery (Moghaddam et al., 2020[33]). Futher, normal levels of selenoprotein P, together with zinc, indicated high chances of survival in COVID-19 patients (Heller et al., 2020[14]). A low-molecular-weight organo selenium drug, Ebselen, has been identified as a potent inhibitor of papain-like protease (PL pro CoV2) which is required for the replication of the virus in the host cell (Węglarz-Tomczak et al., 2020[55]). Papain-like protease (PL pro) was identified as the key enzyme in the pathogenesis of SARS CoV where it aids in viral replication by assembling the new viral particles in the human cells (Báez-Santos et al., 2015[2]). PL pro is also claimed to be an essential viral enzyme that is responsible for the weakening of the antiviral immune response and takes the advantage of the host's immune system for its own benefit (Shin et al., 2020[46]). Selenoproteins, especially glutathione peroxidase (GPx) family, have a major role in regulating the biologic oxidative environment in the cell. In selenium deficient host environment, the decreased activity of these antioxidant enzymes leads to an aggravation of oxidative stress that induces higher mutation rates in viruses. A comparison of sequences of animal coronavirus isolated from selenium-deficient Hubei region and selenium adequate Guangdong region has shown that the virus isolated from Hubei is closely related to the human SARS-CoV virus further indicating the possible role of selenium status in the emergence of new mutations in viral infections (Liu et al., 2007[27]). Recent research using protease cleavage site prediction tools has also predicted the targeting of different selenoproteins viz. Selenoprotein F, thioredoxin reductase 1 and Glutathione peroxidase 1 by the cysteine protease of SARS-CoV-2 (Taylor and Radding, 2020[51]; Seale et al., 2020[45]). Collectively, the emerging evidences in COVID-19 reiterate the crucial role selenium and selenoproteins may have in the pathophysiology of COVID-19 infection.

## Future Perspectives

Computational tools would allow us to identify potential selenoproteins that can be encoded by the COVID-19 virus by the presence of UGA stop codon and secondary structural selenocysteine insertion sequence (SECIS) which occurs immediately after UGA. The mining of these potential virally encoded selenoproteins will facilitate its identification and extraction from biological systems. The association of these potential virally encoded selenoproteins with disease phenotype may give more insights into the biological role of virally encoded selenoproteins in the pathophysiology of COVID-19. Further, it may also help in understanding the effect of an apparent deficiency of selenium created in the host cell by the increased translation of viral selenoproteins on the host cell defense mechanisms against the COVID-19 infection. Since there is unequivocal proof of the involvement of selenium with antioxidant mechanisms in the body, trials of selenium in the treatment of COVID-19 will offer newer opportunities for the treatment of COVID-19. In accordance with the aforementioned role of selenium, improvement in the antioxidant capacity of lungs in ARDS patients has been demonstrated with intravenous sodium selenite infusion (Mahmoodpoor et al., 2019[30]). But further randomized control trials are required to assess the rationale of selenium supplementation in ARDS patients with different etiology viz. viral infections, bacterial infections, or exposure to toxic chemicals.

## Conflict of interest

None declared.

## Research funding

None declared.
